# Assessment of mental health and psychosocial support in Ukrainian refugee minors resettled in Norway

**DOI:** 10.1093/eurpub/ckac130.172

**Published:** 2022-10-25

**Authors:** C Dangmann, M Leonhard, AL Kleppang, L Lien

**Affiliations:** 1 Faculty of Social and Health Sciences, Inland Norway University of Applied Sciences, Elverum, Norway; 2 Advisory Unit Substance Abuse, Mental Health, Innlandet Hospital Trust, Brumunddal, Norway

## Abstract

**Background:**

The recent invasion of Ukraine has forced millions of civilians, especially women and children, to leave their country. Although the European Union offers guidance on individual health assessment of refugees fleeing the war in Ukraine, assessment practice varies across host countries and even on national basis. Thus, the aim of this project was to identify and prioritize procedures for mental health assessment of Ukrainian refugee minors in Norway.

**Methods:**

This project applied a modified three-round-Delphi method. In a first step, the leading public health nurse and community physician in 40 municipalities across Norway were contacted via e-mail and asked to state who is in charge of health assessment, what is current assessing practice and what are the problems and needs. Answers were analysed and condensed and will be presented for rating in a second and third round.

**Results:**

Preliminary results from the first round suggest that most municipalities are currently in a planning phase with uncertainties surrounding who and how future assessments will be done. Public health nurses or general practitioners are often in charge of health assessments, but it is unclear if this includes age-adjusted mental health assessments.

**Conclusions:**

Preliminary results show that current practice in assessing mental health and psychosocial support for Ukranian refugee minors in Norway is very diverse. There is a need to evaluate and prioritize current procedures to assure an equal and age-adjusted procedures for all refugee minors, regardless of where they have resettled.

**Key messages:**

- Current practice in assessing mental health and psychosocial support among Ukrainian refugee minors is very diverse.
- Assessment of mental health in Ukrainian refugees depends on infrastructure and local municipal resources.

